# 1-Ethyl-2-tosyl-4,4,6-trimethyl-2,3,3a,4-tetra­hydro-1*H*-pyrrolo[3,4-*c*]pyrano[6,5-*b*]quinoline-11(6*H*)-one monohydrate

**DOI:** 10.1107/S1600536809030761

**Published:** 2009-08-08

**Authors:** K. Chinnakali, D. Sudha, M. Jayagobi, R. Raghunathan, Hoong-Kun Fun

**Affiliations:** a Department of Physics, Anna University Chennai, Chennai 600 025, India; b Department of Organic Chemistry, University of Madras, Guindy Campus, Chennai 600 025, India; c X-ray Crystallography Unit, School of Physics, Universiti Sains Malaysia, 11800 USM, Penang, Malaysia

## Abstract

In the title compound, C_26_H_30_N_2_O_4_S·H_2_O, the pyrrolidine and dihydro­pyran rings adopt envelope conformations and they are *cis*-fused. The sulfonyl group has a distorted tetra­hedral geometry. In the crystal structure, the mol­ecules are linked into a ribbon-like structure along the *a* axis by O/C—H⋯O hydrogen bonds involving water mol­ecules and C—H⋯π inter­actions involving the sulfonyl-bound phenyl ring. Adjacent ribbons are cross-linked *via* C—H⋯O hydrogen bonds involving a sulfonyl O atom and C—H⋯π inter­actions involving the pyridinone ring.

## Related literature

For the biological activity of pyran­oquinolinones, see: Duraipandiyan & Ignacimuthu (2009 ▶); Magedov *et al.* (2008 ▶); Marco-Contelles *et al.* (2006 ▶). For ring puckering parameters, see: Cremer & Pople (1975 ▶). For asymmetry parameters, see: Duax *et al.* (1976 ▶). For a related structure, see: Chinnakali *et al.* (2007 ▶).
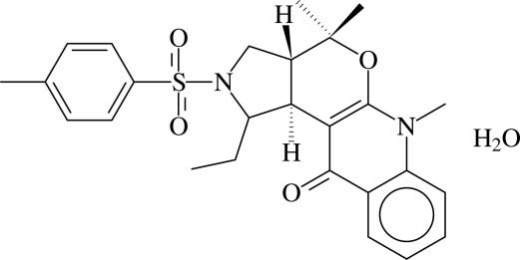


## Experimental

### 

#### Crystal data

C_26_H_30_N_2_O_4_S·H_2_O
*M* *_r_* = 484.60
Triclinic, 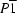

*a* = 9.6964 (2) Å
*b* = 10.2315 (3) Å
*c* = 13.5500 (3) Å
α = 92.143 (1)°
β = 93.142 (1)°
γ = 115.703 (1)°
*V* = 1206.65 (5) Å^3^
*Z* = 2
Mo *K*α radiation
μ = 0.17 mm^−1^
*T* = 100 K
0.58 × 0.32 × 0.32 mm

#### Data collection

Bruker SMART APEXII CCD area-detector diffractometer
Absorption correction: multi-scan (*SADABS*; Bruker, 2005 ▶) *T*_min_ = 0.842, *T*_max_ = 0.947
32886 measured reflections
10512 independent reflections
9074 reflections with *I* > 2σ(*I*)
*R*_int_ = 0.022

#### Refinement

*R*[*F* ^2^ > 2σ(*F* ^2^)] = 0.036
*wR*(*F* ^2^) = 0.108
*S* = 1.04
10512 reflections
320 parameters
H atoms treated by a mixture of independent and constrained refinement
Δρ_max_ = 0.57 e Å^−3^
Δρ_min_ = −0.39 e Å^−3^

### 

Data collection: *APEX2* (Bruker, 2005 ▶); cell refinement: *SAINT* (Bruker, 2005 ▶); data reduction: *SAINT*; program(s) used to solve structure: *SHELXTL* (Sheldrick, 2008 ▶); program(s) used to refine structure: *SHELXTL*; molecular graphics: *SHELXTL*; software used to prepare material for publication: *SHELXTL* and *PLATON* (Spek, 2009 ▶).

## Supplementary Material

Crystal structure: contains datablocks global, I. DOI: 10.1107/S1600536809030761/bq2154sup1.cif

Structure factors: contains datablocks I. DOI: 10.1107/S1600536809030761/bq2154Isup2.hkl

Additional supplementary materials:  crystallographic information; 3D view; checkCIF report

## Figures and Tables

**Table 1:** Hydrogen-bond geometry (Å, °)

*D*—H⋯*A*	*D*—H	H⋯*A*	*D*⋯*A*	*D*—H⋯*A*
O1*W*—H1*W*1⋯O4^i^	0.94 (2)	1.86 (2)	2.7880 (10)	173 (2)
O1*W*—H2*W*1⋯O4	0.94 (2)	1.89 (2)	2.8247 (9)	171 (2)
C24—H24*A*⋯O4	0.97	2.31	3.0213 (11)	129
C24—H24*B*⋯O2	0.97	2.49	3.0748 (11)	119
C15—H15*B*⋯O1^ii^	0.96	2.42	3.3641 (11)	168
C26—H26*C*⋯O1*W*^iii^	0.96	2.45	3.3333 (12)	152
C19—H19⋯*Cg*1^iii^	0.93	2.84	3.6777 (10)	151
C25—H25*B*⋯*Cg*2^iv^	0.96	2.79	3.5358 (12)	135

Symmetry codes: (i) 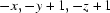; (ii) 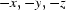; (iii) 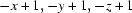; (iv) 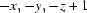. *Cg*1 and *Cg*2 are the centroids of the C8–C13 and N2/C6/C7/C23/C22/C17 rings, respectively.

**Fig. 1.**
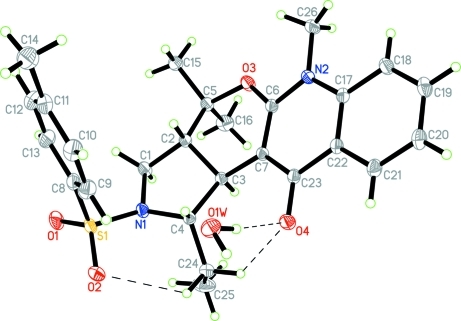
The molecular structure of the title compound. Displacement ellipsoids are drawn at the 50% probability level. Dashed lines indicate hydrogen bonds.

**Fig. 2.**
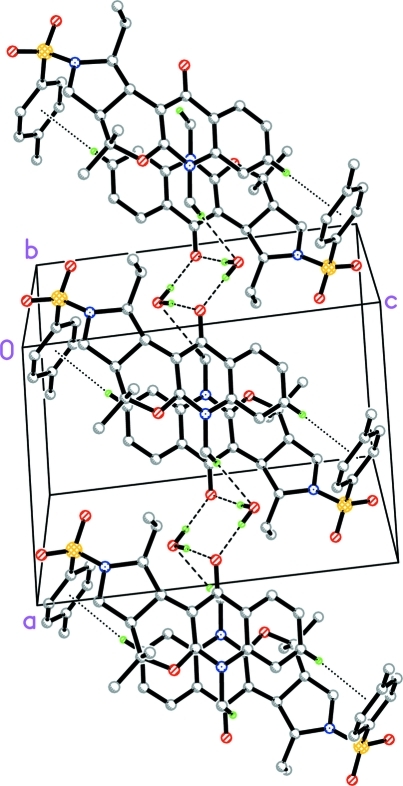
View of a hydrogen-bonded ribbon in the title compound. Dashed and dotted lines indicate O/C—H···O and C—H···π interactions, respectively. For the sake of clarity, H atoms not involved in the interactions have been omitted.

## supplementary crystallographic information

### Comment

Compounds containg pyranoquinolone motifs exhibit antiproliferative and antitubulin activities (Magedov *et al.*, 2008) and antibacterial and antifungal activities (Duraipandiyan & Ignacimuthu, 2009). Some of the pyranoquinoline derivatives have been found to block acetylcholinesterase and cell calcium signals, and cause neuroprotection against calcium overload and free radicals (Marco-Contelles *et al.*, 2006). We report here the crystal structure of the title compound, a pyranoquinolinone derivative.

Bond lengths and angles in the title molecule (Fig. 1) are comparable to those observed in a related compound, *trans*-1-ethyl-4,4,10-trimethyl-2-tosyl- 1,2,3,3a,4,11*b*-hexahydro-11*H*-pyrrolo[3,4-*c*]pyrano[5,6-*c*]quinolin-11-one (Chinnakali *et al.*, 2007). The pyrrolidine ring adopts an envelope conformation with C2, the envelope flap, lying 0.627 (1) Å from the plane defined by atoms N1, C1, C3 and C4. The asymmetry parameter (Duax *et al.*, 1976) ΔC_s_[C2] is 5.85 (7)°, and puckering parameters (Cremer & Pople, 1975) q_2_ and φ are 0.4147 (8) Å and 79.52 (11)°, respectively. The tosyl group is attached to the pyrrolidine ring in a biaxial position. The dihydropyran ring also adopts an envelope conformation, with atom C2 0.694 (1) Å out of the plane formed by the rest of the atoms of the ring. The smallest displacement asymmetry parameter ΔC_s_[C2] is 5.21 (7)°. The pyrrolidine ring is *trans*-fused to the dihydropyran ring. The quinoline ring system is planar (r.m.s. deviation 0.030 Å) with atoms O4 and C26 deviating from the mean plane by 0.043 (1) and 0.083 (1) Å, respectively. The sulfonyl group has a distorted tetrahedral geometry, with the O1—S1—O2 [120.48 (4)°] angle deviating significantly from ideal tetrahedral value. Intramolecular C—H···O hydrogen bonds generate S(6) and S(7) ring motifs.

In the crystal structure, centrosymmetrically related molecules are linked into a dimer by a pair of weak C—H···π interactions (Table 1) involving C19—H19 group and C18—C13 benzene ring (centroid *Cg*1). The dimers are linked into a ribbon like structure along the *a* axis (Fig. 2) by O—H···O and C—H···O hydrogen bonds involving the water molecules. The adjacent ribbons are cross-linked *via* C—H···O hydrogen bonds involving a sulfonyl O atom and C—H···π interactions involving the pyridinone ring.

### Experimental

To a solution of 4-hydroxy-1-methylquinoline (1 mmol) in toluene (20 ml), the corresponding 2-(*N*-prenyl-*N*-tosylamino)acetaldehyde (1 mmol) and a catalytic amount of the base ethylenediamine-*N*,*N*'-diacetate (EDDA, 1 mmol) were added and the reaction mixture was refluxed for 12 h. After completion of the reaction, the solvent was evaporated under reduced pressure and the residue was subjected to column chromatography using a hexane-ethyl acetate (8:2 *v*/*v*) mixture to obtain the title compound. The compound was recrystallized from ethyl acetate solution by slow evaporation.

### Refinement

Water H atoms were located in a difference map and refined freely. The remaining H atoms were positioned geometrically and allowed to ride on their parent atoms, with C—H = 0.93–0.98 Å and *U*_iso_(H) = 1.2–1.5(methyl) *U*_eq_(C). A rotating group model was used for the methyl groups.

### Crystal data

**Table:**

C_26_H_30_N_2_O_4_S·H_2_O	*Z* = 2
*M**_r_* = 484.60	*F*(000) = 516
Triclinic, *P*1	*D*_x_ = 1.334 Mg m^−^^3^
Hall symbol: -P 1	Mo *K*α radiation, λ = 0.71073 Å
*a* = 9.6964 (2) Å	Cell parameters from 9831 reflections
*b* = 10.2315 (3) Å	θ = 2.4–40.2°
*c* = 13.5500 (3) Å	µ = 0.17 mm^−^^1^
α = 92.143 (1)°	*T* = 100 K
β = 93.142 (1)°	Block, colourless
γ = 115.703 (1)°	0.58 × 0.32 × 0.32 mm
*V* = 1206.65 (5) Å^3^	

### Data collection

**Table:**

Bruker SMART APEXII CCD area-detector diffractometer	10512 independent reflections
Radiation source: fine-focus sealed tube	9074 reflections with *I* > 2σ(*I*)
graphite	*R*_int_ = 0.022
φ and ω scans	θ_max_ = 35.0°, θ_min_ = 2.3°
Absorption correction: multi-scan (*SADABS*; Bruker, 2005)	*h* = −14→15
*T*_min_ = 0.842, *T*_max_ = 0.947	*k* = −16→16
32886 measured reflections	*l* = −21→21

### Refinement

**Table:**

Refinement on *F*^2^	Primary atom site location: structure-invariant direct methods
Least-squares matrix: full	Secondary atom site location: difference Fourier map
*R*[*F*^2^ > 2σ(*F*^2^)] = 0.036	Hydrogen site location: inferred from neighbouring sites
*wR*(*F*^2^) = 0.108	H atoms treated by a mixture of independent and constrained refinement
*S* = 1.03	*w* = 1/[σ^2^(*F*_o_^2^) + (0.0587*P*)^2^ + 0.2725*P*] where *P* = (*F*_o_^2^ + 2*F*_c_^2^)/3
10512 reflections	(Δ/σ)_max_ = 0.001
320 parameters	Δρ_max_ = 0.57 e Å^−^^3^
0 restraints	Δρ_min_ = −0.39 e Å^−^^3^

### Special details

**Table:**

Geometry. All e.s.d.'s (except the e.s.d. in the dihedral angle between two l.s. planes) are estimated using the full covariance matrix. The cell e.s.d.'s are taken into account individually in the estimation of e.s.d.'s in distances, angles and torsion angles; correlations between e.s.d.'s in cell parameters are only used when they are defined by crystal symmetry. An approximate (isotropic) treatment of cell e.s.d.'s is used for estimating e.s.d.'s involving l.s. planes.
Refinement. Refinement of *F*^2^ against ALL reflections. The weighted *R*-factor *wR* and goodness of fit *S* are based on *F*^2^, conventional *R*-factors *R* are based on *F*, with *F* set to zero for negative *F*^2^. The threshold expression of *F*^2^ > σ(*F*^2^) is used only for calculating *R*-factors(gt) *etc*. and is not relevant to the choice of reflections for refinement. *R*-factors based on *F*^2^ are statistically about twice as large as those based on *F*, and *R*- factors based on ALL data will be even larger.

### Fractional atomic coordinates and isotropic or equivalent isotropic displacement parameters (Å^2^)

**Table:**

	*x*	*y*	*z*	*U*_iso_*/*U*_eq_	
S1	−0.10550 (2)	0.20102 (2)	0.101937 (13)	0.01815 (5)	
O1	−0.14482 (8)	0.10439 (8)	0.01424 (5)	0.02588 (13)	
O2	−0.21397 (7)	0.24814 (8)	0.13802 (5)	0.02463 (12)	
O3	0.36172 (7)	0.10968 (7)	0.36629 (4)	0.02095 (11)	
O4	0.05379 (7)	0.31969 (7)	0.50127 (4)	0.02048 (11)	
N1	−0.06215 (7)	0.11918 (7)	0.19027 (5)	0.01627 (11)	
N2	0.44953 (7)	0.26770 (7)	0.49833 (5)	0.01623 (11)	
C1	0.03179 (9)	0.04091 (8)	0.17054 (6)	0.01816 (12)	
H1A	0.0728	0.0600	0.1062	0.022*	
H1B	−0.0267	−0.0631	0.1744	0.022*	
C2	0.15833 (8)	0.10586 (8)	0.25395 (5)	0.01455 (11)	
H2	0.2276	0.2040	0.2370	0.017*	
C3	0.07378 (8)	0.12244 (7)	0.34232 (5)	0.01382 (11)	
H3	0.0114	0.0254	0.3642	0.017*	
C4	−0.03588 (8)	0.18134 (8)	0.29580 (5)	0.01449 (11)	
H4	0.0166	0.2877	0.2976	0.017*	
C5	0.25658 (8)	0.02781 (8)	0.27888 (5)	0.01649 (12)	
C6	0.32986 (8)	0.19511 (8)	0.42907 (5)	0.01509 (11)	
C7	0.19301 (8)	0.20919 (8)	0.42556 (5)	0.01399 (11)	
C8	0.06168 (9)	0.35827 (9)	0.08100 (5)	0.01895 (13)	
C9	0.09398 (10)	0.48997 (9)	0.13299 (6)	0.02292 (14)	
H9	0.0276	0.4957	0.1781	0.027*	
C10	0.22655 (11)	0.61223 (10)	0.11634 (7)	0.02546 (16)	
H10	0.2482	0.7000	0.1509	0.031*	
C11	0.32828 (10)	0.60689 (10)	0.04910 (6)	0.02372 (15)	
C12	0.29358 (11)	0.47384 (11)	−0.00188 (6)	0.02569 (16)	
H12	0.3601	0.4681	−0.0469	0.031*	
C13	0.16132 (10)	0.34970 (10)	0.01340 (6)	0.02375 (15)	
H13	0.1396	0.2618	−0.0211	0.028*	
C14	0.47103 (12)	0.74153 (12)	0.03266 (8)	0.03231 (19)	
H14A	0.4436	0.8179	0.0156	0.048*	
H14B	0.5221	0.7210	−0.0203	0.048*	
H14C	0.5385	0.7716	0.0922	0.048*	
C15	0.36409 (9)	0.03800 (9)	0.19901 (6)	0.02084 (14)	
H15A	0.4236	0.1382	0.1867	0.031*	
H15B	0.3051	−0.0143	0.1392	0.031*	
H15C	0.4315	−0.0034	0.2207	0.031*	
C16	0.16794 (11)	−0.12767 (9)	0.30705 (7)	0.02468 (15)	
H16A	0.1110	−0.1286	0.3630	0.037*	
H16B	0.2385	−0.1678	0.3238	0.037*	
H16C	0.0984	−0.1849	0.2521	0.037*	
C17	0.43494 (8)	0.35304 (8)	0.57565 (5)	0.01535 (11)	
C18	0.55160 (9)	0.41818 (8)	0.65335 (6)	0.01901 (13)	
H18	0.6413	0.4062	0.6529	0.023*	
C19	0.53158 (10)	0.49978 (9)	0.73000 (6)	0.02148 (14)	
H19	0.6081	0.5415	0.7814	0.026*	
C20	0.39817 (10)	0.52082 (9)	0.73172 (6)	0.02210 (14)	
H20	0.3871	0.5777	0.7830	0.027*	
C21	0.28324 (9)	0.45606 (8)	0.65626 (6)	0.01910 (13)	
H21	0.1941	0.4690	0.6574	0.023*	
C22	0.29908 (8)	0.37079 (8)	0.57767 (5)	0.01509 (11)	
C23	0.17397 (8)	0.30031 (8)	0.50003 (5)	0.01469 (11)	
C24	−0.19016 (9)	0.13330 (9)	0.34164 (6)	0.01990 (13)	
H24A	−0.1706	0.1584	0.4124	0.024*	
H24B	−0.2427	0.1872	0.3142	0.024*	
C25	−0.29541 (11)	−0.02827 (11)	0.32456 (9)	0.0329 (2)	
H25A	−0.3893	−0.0501	0.3551	0.049*	
H25B	−0.2457	−0.0828	0.3531	0.049*	
H25C	−0.3177	−0.0540	0.2547	0.049*	
C26	0.59115 (9)	0.24915 (10)	0.49227 (6)	0.02184 (14)	
H26A	0.6064	0.2347	0.4241	0.033*	
H26B	0.5825	0.1661	0.5270	0.033*	
H26C	0.6769	0.3345	0.5218	0.033*	
O1W	0.07659 (8)	0.54423 (7)	0.37852 (5)	0.02352 (12)	
H1W1	0.039 (2)	0.5967 (19)	0.4179 (14)	0.057 (5)*	
H2W1	0.0587 (19)	0.4646 (19)	0.4169 (13)	0.050 (5)*	

### Atomic displacement parameters (Å^2^)

**Table:**

	*U*^11^	*U*^22^	*U*^33^	*U*^12^	*U*^13^	*U*^23^
S1	0.01901 (8)	0.02528 (9)	0.01322 (8)	0.01303 (7)	−0.00226 (6)	−0.00017 (6)
O1	0.0270 (3)	0.0342 (3)	0.0153 (2)	0.0138 (3)	−0.0065 (2)	−0.0055 (2)
O2	0.0232 (3)	0.0363 (3)	0.0231 (3)	0.0210 (3)	0.0015 (2)	0.0048 (2)
O3	0.0222 (2)	0.0308 (3)	0.0164 (2)	0.0188 (2)	−0.00380 (19)	−0.0072 (2)
O4	0.0218 (2)	0.0277 (3)	0.0182 (2)	0.0173 (2)	−0.00107 (19)	−0.0039 (2)
N1	0.0196 (3)	0.0198 (3)	0.0127 (2)	0.0121 (2)	−0.00137 (19)	−0.00144 (19)
N2	0.0152 (2)	0.0210 (3)	0.0139 (2)	0.0096 (2)	−0.00049 (19)	−0.0008 (2)
C1	0.0211 (3)	0.0210 (3)	0.0157 (3)	0.0133 (3)	−0.0028 (2)	−0.0045 (2)
C2	0.0162 (3)	0.0165 (3)	0.0131 (3)	0.0094 (2)	0.0002 (2)	−0.0011 (2)
C3	0.0146 (3)	0.0156 (3)	0.0129 (3)	0.0084 (2)	0.0002 (2)	−0.0003 (2)
C4	0.0159 (3)	0.0169 (3)	0.0125 (3)	0.0092 (2)	0.0000 (2)	−0.0004 (2)
C5	0.0183 (3)	0.0195 (3)	0.0145 (3)	0.0114 (2)	−0.0008 (2)	−0.0026 (2)
C6	0.0164 (3)	0.0187 (3)	0.0125 (3)	0.0100 (2)	0.0004 (2)	0.0000 (2)
C7	0.0153 (3)	0.0166 (3)	0.0121 (2)	0.0091 (2)	0.0004 (2)	0.0000 (2)
C8	0.0236 (3)	0.0244 (3)	0.0131 (3)	0.0144 (3)	0.0010 (2)	0.0019 (2)
C9	0.0298 (4)	0.0248 (3)	0.0205 (3)	0.0175 (3)	0.0048 (3)	0.0022 (3)
C10	0.0327 (4)	0.0237 (4)	0.0243 (4)	0.0162 (3)	0.0032 (3)	0.0020 (3)
C11	0.0262 (4)	0.0277 (4)	0.0190 (3)	0.0132 (3)	0.0007 (3)	0.0058 (3)
C12	0.0275 (4)	0.0329 (4)	0.0183 (3)	0.0143 (3)	0.0049 (3)	0.0017 (3)
C13	0.0275 (4)	0.0293 (4)	0.0166 (3)	0.0145 (3)	0.0037 (3)	−0.0018 (3)
C14	0.0295 (4)	0.0321 (4)	0.0327 (5)	0.0105 (4)	0.0027 (4)	0.0084 (4)
C15	0.0212 (3)	0.0283 (4)	0.0173 (3)	0.0152 (3)	0.0013 (2)	−0.0035 (3)
C16	0.0272 (4)	0.0210 (3)	0.0313 (4)	0.0152 (3)	0.0039 (3)	0.0051 (3)
C17	0.0167 (3)	0.0160 (3)	0.0129 (3)	0.0068 (2)	0.0006 (2)	0.0014 (2)
C18	0.0179 (3)	0.0199 (3)	0.0165 (3)	0.0062 (2)	−0.0020 (2)	0.0009 (2)
C19	0.0241 (3)	0.0198 (3)	0.0163 (3)	0.0064 (3)	−0.0033 (3)	−0.0011 (2)
C20	0.0283 (4)	0.0217 (3)	0.0154 (3)	0.0107 (3)	−0.0014 (3)	−0.0031 (2)
C21	0.0235 (3)	0.0200 (3)	0.0150 (3)	0.0109 (3)	0.0003 (2)	−0.0019 (2)
C22	0.0179 (3)	0.0159 (3)	0.0122 (3)	0.0082 (2)	0.0004 (2)	0.0002 (2)
C23	0.0178 (3)	0.0164 (3)	0.0121 (3)	0.0096 (2)	0.0009 (2)	0.0007 (2)
C24	0.0169 (3)	0.0283 (4)	0.0175 (3)	0.0126 (3)	0.0023 (2)	0.0020 (3)
C25	0.0183 (3)	0.0290 (4)	0.0507 (6)	0.0084 (3)	0.0073 (4)	0.0134 (4)
C26	0.0175 (3)	0.0314 (4)	0.0204 (3)	0.0147 (3)	−0.0009 (2)	−0.0011 (3)
O1W	0.0259 (3)	0.0248 (3)	0.0238 (3)	0.0147 (2)	0.0038 (2)	−0.0016 (2)

### Geometric parameters (Å, °)

**Table:**

S1—O2	1.4341 (6)	C12—C13	1.3916 (13)
S1—O1	1.4389 (6)	C12—H12	0.93
S1—N1	1.6234 (6)	C13—H13	0.93
S1—C8	1.7661 (8)	C14—H14A	0.96
O3—C6	1.3381 (9)	C14—H14B	0.96
O3—C5	1.4821 (9)	C14—H14C	0.96
O4—C23	1.2648 (9)	C15—H15A	0.96
N1—C1	1.4784 (9)	C15—H15B	0.96
N1—C4	1.5052 (9)	C15—H15C	0.96
N2—C6	1.3637 (9)	C16—H16A	0.96
N2—C17	1.3911 (9)	C16—H16B	0.96
N2—C26	1.4716 (10)	C16—H16C	0.96
C1—C2	1.5196 (10)	C17—C22	1.4071 (10)
C1—H1A	0.97	C17—C18	1.4126 (10)
C1—H1B	0.97	C18—C19	1.3825 (11)
C2—C5	1.5179 (10)	C18—H18	0.93
C2—C3	1.5321 (10)	C19—C20	1.4017 (12)
C2—H2	0.98	C19—H19	0.93
C3—C7	1.5128 (10)	C20—C21	1.3822 (11)
C3—C4	1.5499 (10)	C20—H20	0.93
C3—H3	0.98	C21—C22	1.4084 (10)
C4—C24	1.5337 (10)	C21—H21	0.93
C4—H4	0.98	C22—C23	1.4640 (10)
C5—C15	1.5187 (11)	C24—C25	1.5198 (13)
C5—C16	1.5201 (11)	C24—H24A	0.97
C6—C7	1.3940 (10)	C24—H24B	0.97
C7—C23	1.4216 (10)	C25—H25A	0.96
C8—C13	1.3944 (11)	C25—H25B	0.96
C8—C9	1.3960 (11)	C25—H25C	0.96
C9—C10	1.3878 (13)	C26—H26A	0.96
C9—H9	0.93	C26—H26B	0.96
C10—C11	1.3956 (13)	C26—H26C	0.96
C10—H10	0.93	O1W—H1W1	0.933 (18)
C11—C12	1.3965 (13)	O1W—H2W1	0.940 (18)
C11—C14	1.5053 (13)		
			
O2—S1—O1	120.48 (4)	C11—C12—H12	119.4
O2—S1—N1	106.96 (4)	C12—C13—C8	119.38 (8)
O1—S1—N1	106.25 (4)	C12—C13—H13	120.3
O2—S1—C8	107.39 (4)	C8—C13—H13	120.3
O1—S1—C8	107.16 (4)	C11—C14—H14A	109.5
N1—S1—C8	108.10 (4)	C11—C14—H14B	109.5
C6—O3—C5	122.23 (6)	H14A—C14—H14B	109.5
C1—N1—C4	112.20 (5)	C11—C14—H14C	109.5
C1—N1—S1	119.56 (5)	H14A—C14—H14C	109.5
C4—N1—S1	120.20 (5)	H14B—C14—H14C	109.5
C6—N2—C17	120.21 (6)	C5—C15—H15A	109.5
C6—N2—C26	118.97 (6)	C5—C15—H15B	109.5
C17—N2—C26	120.77 (6)	H15A—C15—H15B	109.5
N1—C1—C2	101.53 (5)	C5—C15—H15C	109.5
N1—C1—H1A	111.5	H15A—C15—H15C	109.5
C2—C1—H1A	111.5	H15B—C15—H15C	109.5
N1—C1—H1B	111.5	C5—C16—H16A	109.5
C2—C1—H1B	111.5	C5—C16—H16B	109.5
H1A—C1—H1B	109.3	H16A—C16—H16B	109.5
C5—C2—C1	118.88 (6)	C5—C16—H16C	109.5
C5—C2—C3	112.23 (6)	H16A—C16—H16C	109.5
C1—C2—C3	103.40 (6)	H16B—C16—H16C	109.5
C5—C2—H2	107.2	N2—C17—C22	119.09 (6)
C1—C2—H2	107.2	N2—C17—C18	121.27 (7)
C3—C2—H2	107.2	C22—C17—C18	119.61 (7)
C7—C3—C2	107.88 (5)	C19—C18—C17	119.61 (7)
C7—C3—C4	120.70 (6)	C19—C18—H18	120.2
C2—C3—C4	102.90 (5)	C17—C18—H18	120.2
C7—C3—H3	108.2	C18—C19—C20	121.29 (7)
C2—C3—H3	108.2	C18—C19—H19	119.4
C4—C3—H3	108.2	C20—C19—H19	119.4
N1—C4—C24	109.79 (6)	C21—C20—C19	119.20 (7)
N1—C4—C3	102.05 (5)	C21—C20—H20	120.4
C24—C4—C3	115.58 (6)	C19—C20—H20	120.4
N1—C4—H4	109.7	C20—C21—C22	120.98 (7)
C24—C4—H4	109.7	C20—C21—H21	119.5
C3—C4—H4	109.7	C22—C21—H21	119.5
O3—C5—C2	106.88 (6)	C17—C22—C21	119.29 (7)
O3—C5—C15	103.73 (6)	C17—C22—C23	120.83 (6)
C2—C5—C15	112.07 (6)	C21—C22—C23	119.88 (7)
O3—C5—C16	106.72 (6)	O4—C23—C7	122.29 (7)
C2—C5—C16	114.79 (6)	O4—C23—C22	120.33 (6)
C15—C5—C16	111.75 (6)	C7—C23—C22	117.37 (6)
O3—C6—N2	111.07 (6)	C25—C24—C4	114.19 (7)
O3—C6—C7	125.22 (6)	C25—C24—H24A	108.7
N2—C6—C7	123.71 (6)	C4—C24—H24A	108.7
C6—C7—C23	118.59 (6)	C25—C24—H24B	108.7
C6—C7—C3	116.49 (6)	C4—C24—H24B	108.7
C23—C7—C3	124.90 (6)	H24A—C24—H24B	107.6
C13—C8—C9	120.45 (8)	C24—C25—H25A	109.5
C13—C8—S1	119.77 (6)	C24—C25—H25B	109.5
C9—C8—S1	119.77 (6)	H25A—C25—H25B	109.5
C10—C9—C8	119.05 (8)	C24—C25—H25C	109.5
C10—C9—H9	120.5	H25A—C25—H25C	109.5
C8—C9—H9	120.5	H25B—C25—H25C	109.5
C9—C10—C11	121.74 (8)	N2—C26—H26A	109.5
C9—C10—H10	119.1	N2—C26—H26B	109.5
C11—C10—H10	119.1	H26A—C26—H26B	109.5
C10—C11—C12	118.13 (8)	N2—C26—H26C	109.5
C10—C11—C14	120.43 (9)	H26A—C26—H26C	109.5
C12—C11—C14	121.44 (8)	H26B—C26—H26C	109.5
C13—C12—C11	121.24 (8)	H1W1—O1W—H2W1	100.8 (14)
C13—C12—H12	119.4		
			
O2—S1—N1—C1	171.79 (6)	C2—C3—C7—C23	151.18 (7)
O1—S1—N1—C1	41.91 (7)	C4—C3—C7—C23	33.50 (10)
C8—S1—N1—C1	−72.84 (6)	O2—S1—C8—C13	−155.91 (7)
O2—S1—N1—C4	−41.83 (7)	O1—S1—C8—C13	−25.14 (8)
O1—S1—N1—C4	−171.71 (6)	N1—S1—C8—C13	89.01 (7)
C8—S1—N1—C4	73.54 (6)	O2—S1—C8—C9	24.75 (8)
C4—N1—C1—C2	−20.95 (8)	O1—S1—C8—C9	155.53 (7)
S1—N1—C1—C2	127.93 (6)	N1—S1—C8—C9	−90.33 (7)
N1—C1—C2—C5	163.47 (6)	C13—C8—C9—C10	0.08 (12)
N1—C1—C2—C3	38.34 (7)	S1—C8—C9—C10	179.42 (7)
C5—C2—C3—C7	59.85 (7)	C8—C9—C10—C11	−0.04 (13)
C1—C2—C3—C7	−170.83 (6)	C9—C10—C11—C12	−0.07 (13)
C5—C2—C3—C4	−171.52 (6)	C9—C10—C11—C14	179.94 (8)
C1—C2—C3—C4	−42.20 (7)	C10—C11—C12—C13	0.14 (13)
C1—N1—C4—C24	−127.76 (7)	C14—C11—C12—C13	−179.87 (8)
S1—N1—C4—C24	83.59 (7)	C11—C12—C13—C8	−0.10 (13)
C1—N1—C4—C3	−4.66 (8)	C9—C8—C13—C12	−0.02 (13)
S1—N1—C4—C3	−153.32 (5)	S1—C8—C13—C12	−179.35 (7)
C7—C3—C4—N1	148.43 (6)	C6—N2—C17—C22	−4.08 (10)
C2—C3—C4—N1	28.27 (7)	C26—N2—C17—C22	178.58 (7)
C7—C3—C4—C24	−92.49 (8)	C6—N2—C17—C18	173.96 (7)
C2—C3—C4—C24	147.35 (6)	C26—N2—C17—C18	−3.38 (11)
C6—O3—C5—C2	23.11 (9)	N2—C17—C18—C19	−178.65 (7)
C6—O3—C5—C15	141.69 (7)	C22—C17—C18—C19	−0.62 (11)
C6—O3—C5—C16	−100.17 (8)	C17—C18—C19—C20	−0.76 (12)
C1—C2—C5—O3	−176.29 (6)	C18—C19—C20—C21	1.36 (12)
C3—C2—C5—O3	−55.54 (8)	C19—C20—C21—C22	−0.58 (12)
C1—C2—C5—C15	70.71 (9)	N2—C17—C22—C21	179.45 (7)
C3—C2—C5—C15	−168.54 (6)	C18—C17—C22—C21	1.38 (10)
C1—C2—C5—C16	−58.17 (9)	N2—C17—C22—C23	0.26 (10)
C3—C2—C5—C16	62.58 (8)	C18—C17—C22—C23	−177.81 (7)
C5—O3—C6—N2	−174.90 (6)	C20—C21—C22—C17	−0.78 (11)
C5—O3—C6—C7	5.28 (11)	C20—C21—C22—C23	178.41 (7)
C17—N2—C6—O3	−174.60 (6)	C6—C7—C23—O4	179.29 (7)
C26—N2—C6—O3	2.79 (10)	C3—C7—C23—O4	−1.91 (11)
C17—N2—C6—C7	5.22 (11)	C6—C7—C23—C22	−1.63 (10)
C26—N2—C6—C7	−177.39 (7)	C3—C7—C23—C22	177.18 (6)
O3—C6—C7—C23	177.57 (7)	C17—C22—C23—O4	−178.34 (7)
N2—C6—C7—C23	−2.22 (11)	C21—C22—C23—O4	2.47 (11)
O3—C6—C7—C3	−1.33 (11)	C17—C22—C23—C7	2.55 (10)
N2—C6—C7—C3	178.88 (6)	C21—C22—C23—C7	−176.63 (7)
C2—C3—C7—C6	−30.00 (8)	N1—C4—C24—C25	47.81 (9)
C4—C3—C7—C6	−147.68 (7)	C3—C4—C24—C25	−66.92 (9)

### Hydrogen-bond geometry (Å, °)

**Table:**

*D*—H···*A*	*D*—H	H···*A*	*D*···*A*	*D*—H···*A*
O1W—H1W1···O4^i^	0.94 (2)	1.86 (2)	2.7880 (10)	173 (2)
O1W—H2W1···O4	0.94 (2)	1.89 (2)	2.8247 (9)	171 (2)
C24—H24A···O4	0.97	2.31	3.0213 (11)	129
C24—H24B···O2	0.97	2.49	3.0748 (11)	119
C15—H15B···O1^ii^	0.96	2.42	3.3641 (11)	168
C26—H26C···O1W^iii^	0.96	2.45	3.3333 (12)	152
C19—H19···Cg1^iii^	0.93	2.84	3.6777 (10)	151
C25—H25B···Cg2^iv^	0.96	2.79	3.5358 (12)	135

Symmetry codes: (i) −*x*, −*y*+1, −*z*+1; (ii) −*x*, −*y*, −*z*; (iii) −*x*+1, −*y*+1, −*z*+1; (iv) −*x*, −*y*, −*z*+1.
